# Direct Evidence for a Peroxide Intermediate and a Reactive Enzyme–Substrate–Dioxygen Configuration in a Cofactor-free Oxidase**

**DOI:** 10.1002/anie.201405485

**Published:** 2014-10-14

**Authors:** Soi Bui, David von Stetten, Pablo G Jambrina, Thierry Prangé, Nathalie Colloc'h, Daniele de Sanctis, Antoine Royant, Edina Rosta, Roberto A Steiner

**Affiliations:** Randall Division of Cell and Molecular Biophysics, King's College LondonNew Hunt's House, Guy's Campus, London SE1 1UL (UK); European Synchrotron Radiation FacilityCS 40220, 38043 Grenoble Cedex 9 (France); Department of Chemistry, King's College LondonBritannia House 7 Trinity Street, London, SE1 1DB (UK); LCRB, UMR 8015-Université Paris Descartes-CNRSFaculté de Pharmacie 75270 Paris Cedex 06 (France); ISTCT, UMR 6301-UCBN-CNRS-CEA-Normandie UniversitéCentre Cyceron, 14074 Caen Cedex (France); Institut de Biologie StructuraleUMR 5075 Université Grenoble Alpes-CNRS-CEA, CS10090, 38044 Grenoble Cedex 9 (France)

**Keywords:** dioxygen, enzymatic mechanisms, oxidases, reaction intermediates, structural enzymology

## Abstract

Cofactor-free oxidases and oxygenases promote and control the reactivity of O_2_ with limited chemical tools at their disposal. Their mechanism of action is not completely understood and structural information is not available for any of the reaction intermediates. Near-atomic resolution crystallography supported by in crystallo Raman spectroscopy and QM/MM calculations showed unambiguously that the archetypical cofactor-free uricase catalyzes uric acid degradation via a C5(*S*)-(hydro)peroxide intermediate. Low X-ray doses break specifically the intermediate C5=OO(H) bond at 100 K, thus releasing O_2_ in situ, which is trapped above the substrate radical. The dose-dependent rate of bond rupture followed by combined crystallographic and Raman analysis indicates that ionizing radiation kick-starts both peroxide decomposition and its regeneration. Peroxidation can be explained by a mechanism in which the substrate radical recombines with superoxide transiently produced in the active site.

The majority of enzymes that use dioxygen as a reactant require metal or organic cofactors for catalysis. Cofactors are typically needed to engender organic radicals that can react directly with O_2_ or elicit its activation, for example, to a superoxide anion. A number of oxidases and oxygenases, however, operate in a cofactor-free manner, relying on very limited chemical tools to promote and control O_2_ chemistry.[1] At present, no structural information is available for any of the reaction intermediates in this enzyme class.

The archetypal cofactor-free oxidase is uricase (urate oxidase or rasburicase, UOX), a long-investigated, therapeutically important (commercialized as Elitek in the US and Fasturtec in Europe), tetrameric 133 kDa enzyme that catalyzes the O_2_-mediated degradation of uric acid (UA) to 5-hydroxyisourate (5-HIU, Figure 1 a, see also the Supporting Information, Figure S1).[2] Similarly to flavin-dependent monooxygenases that operate via a C4a-(hydro)peroxyflavin intermediate,[3] a reaction pathway has been proposed for UOX that involves 5-peroxyisourate (5-PIU) generated by the recombination of urate radical and superoxide anion (path A in Figure 1 b).[2c,d] A mechanism involving a peroxide has also been proposed for bacterial cofactor-independent dioxygenases.[4] Other studies have put forward a UOX peroxide-independent mechanism[5] (path B in Figure 1 b) akin to that of flavin-dependent oxidases.[3]

**Figure 1:**
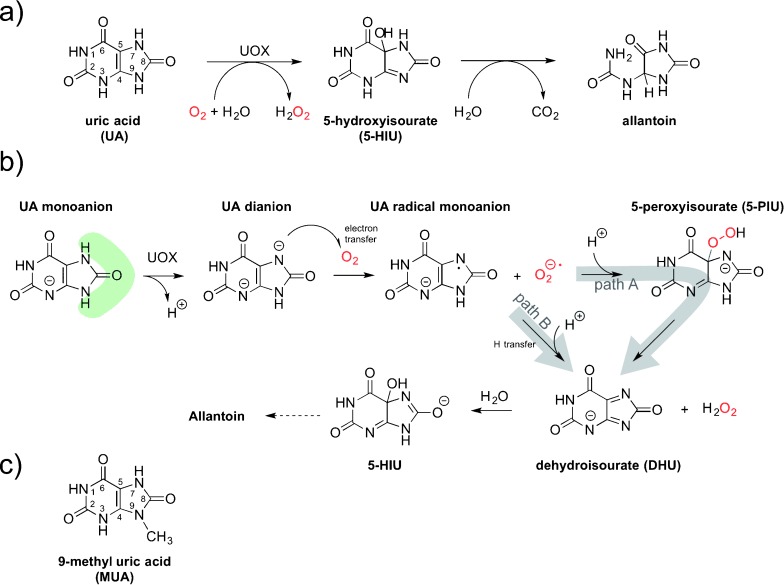
UOX-catalyzed reaction, mechanistic proposals, and alternative substrate. a) UOX degrades UA to 5-HIU. In solution, 5-HIU evolves spontaneously to a racemic mixture of allantoin, while in vivo it is rapidly degraded to (+)-allantoin by a separate enzyme system.[15] b) Peroxide-dependent[2d, 16] (path A) and peroxide-independent[5] (path B) mechanistic proposals. The shaded green area emphasizes the solvent network surrounding the five-membered ring. c) Chemical structure of MUA used in this study.

To investigate the reaction steps involving dioxygen in UOX-mediated catalysis, we employed 9-methyl uric acid (MUA, Figure 1 c) as we reasoned that methyl substitution at position 9 could stabilize the putative peroxide intermediate. Crystallographic analysis at near-atomic resolution (see Table S1 for data collection and refinement statistics) showed that under strict anaerobic conditions, MUA binds, as do UA[6] and several inhibitors,[2a, 7] at the interface between two UOX protomers in a 35 Å^3^ cavity exposed to the solvent on the side of the substrate’s imidazole ring (Figure 2 a). A combination of hydrogen bonds, salt bridges, and hydrophobic interactions holds the flat MUA in place. A water molecule (W1) is held above the C4=C5 bond at a distance of 3.0 Å from the MUA plane by H-bonds with T57(Oγ1) and N254(Nδ2). An additional water molecule (W2), part of a complex solvent network, interacts with the O8 atom of the substrate. When anaerobic UOX:MUA co-crystals were exposed to air (or alternatively O_2_), electron-density maps at 1.32 Å unambiguously showed that MUA converts into its C5(*S*)-peroxo derivative (5-PMUA, 9-methyl-5-PIU) with clear electron density for the Op1 and Op2 peroxide oxygen atoms (Figure 2 b, Figure S2). 5-PMUA is pyramidalized at carbon atom C5 with tetrahedral angles ranging from 94.4° (C6-C5-Op1) to 121.5° (C6-C5-N7). The rather acute C6-C5-Op1 angle results in the newly formed C5=Op1 bond leaning toward the pyrimidine ring. Its bond length refines to 1.51 Å, while the peroxo Op1=Op2 bond is 1.47 Å long. Quantum mechanics/molecular mechanics (QM/MM) calculations suggested that the experimental C5=Op1 distance is most consistent with a hydroperoxide monoanion (see Table S2). The Op2 (hydro)peroxo atom is asymmetrically H-bonded to T57(Oγ1) and N254(Nδ2) at 2.63 Å and 3.10 Å, respectively, with the C6-C5-Op1-Op2 torsion angle measuring 175.5°, thus indicating that the projection of the Op1=Op2 bond is essentially aligned to C5=C6 of the six-membered ring. The distortion of the purine ring following peroxidation does not alter the H-bond/salt bridge interactions seen within the catalytic pocket for the enzyme:substrate complex (Figure 2 a), thus suggesting that the active site is pre-organized to accommodate reactants and intermediates with very limited protein rearrangements. Instead, a reconfiguration of the solvent takes place. Upon peroxidation, water (W2) moves away from the O8 atom of the substrate at a distance too long for an H-bond interaction (>4.5 Å).

**Figure 2:**
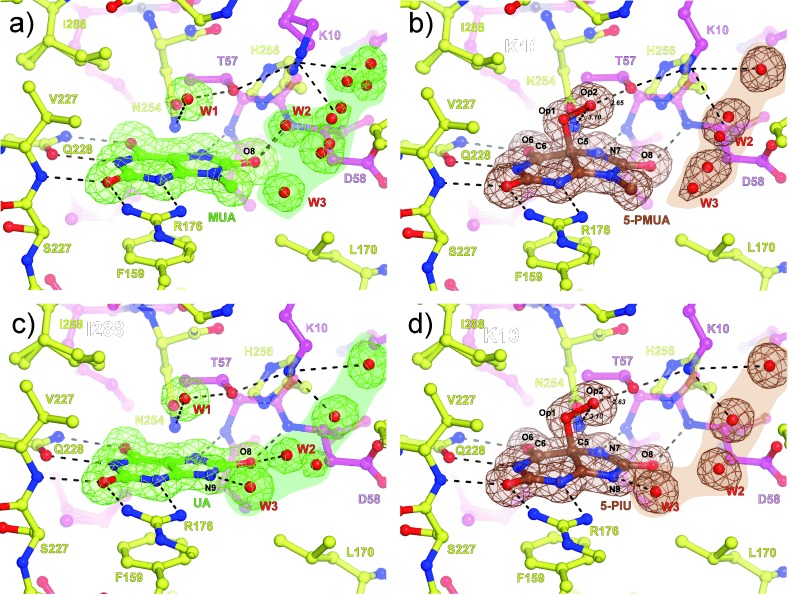
UOX-mediated catalysis proceeding via a (hydro)peroxide intermediate. a) Anaerobic UOX:MUA complex. b) UOX:5-PMUA complex. c) Anaerobic UOX:UA complex. d) UOX:5-PIU complex. UOX residues at the interface are shown in stick representation and are color-coded according to the subunit they belong to. Red spheres represent water molecules. In (a,c), 2 *m* *F*_o_−*D* *F*_c_ electron density contoured at 1σ level is shown in green for the bound MUA and UA as well for the solvent molecules in their proximity. In (b,d), 2 *m* *F*_o_−*D* *F*_c_ electron density contoured at 1σ level is shown in brown for the peroxides and solvent network. Hydrogen bonds are shown as black dashed lines. The shaded regions highlight the solvent pool contributed by several water molecules connected by H-bonds (not shown for clarity) often exhibiting partial occupancy. In the anaerobic samples, a water molecule (W2) is H-bonded to the O8 atom of the substrate. This interaction is lost upon peroxide formation. W1 is displaced upon peroxide formation. Op1 and Op2 indicate peroxide oxygens.

Visualization of 5-PMUA in the crystal prompted us to attempt the trapping of the 5-PIU peroxo intermediate using UA, UOX’s natural substrate. UOX crystals cannot be incubated with UA under aerobic conditions, as this leads to degradation to allantoin.[8] We thus soaked anaerobic UOX:UA crystals (Figure 2 c) in a reservoir spiked with H_2_O_2_ to favor an enrichment of the peroxide population as a result of the O_2_/H_2_O_2_ equilibrium and the law of mass action. This approach led to the trapping of the UOX:5-PIU complex at 1.3 Å (Figure 2 d). 5-PIU and 5-PMUA geometries are essentially identical (see Table S2). Our results unambiguously show that the UOX reaction proceeds via the C5-peroxide intermediate. Peroxide intermediacy establishes a mechanistic link between cofactor-independent UOX and flavin-dependent monooxigenases.[3] For the latter enzyme class, the C4a-(hydro)peroxyflavin intermediate, although well-characterized spectroscopically, has never been structurally determined.

To gain further insight into the enzyme:peroxide complex, we employed in crystallo Raman micro-spectrophotometry complemented by theoretical methods. Nonresonant Raman spectra recorded from crystals of substrate-free UOX, anaerobic UOX:MUA, and UOX:5-PMUA complexes are very similar overall (Figure 3 a). However, the spectral region centered at 600 cm^−1^ shows changes sensitive to the chemistry of the process (Figure 3 b). Upon MUA peroxidation, a distinct band develops at 605 cm^−1^ (brown trace), while the shoulder at 597 cm^−1^ in the UOX:MUA complex (green) disappears. Neither band is present in the spectrum of substrate-free UOX (grey), thus indicating that they arise from the bound organic molecules. Furthermore, the 605 cm^−1^ band could be selectively isotopically shifted by carrying out MUA peroxidation with ^18^O_2_ (shift to 596 cm^−1^, 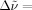
−9 cm^−1^, orange), thus confirming that this band specifically involves Raman modes with contributions from the peroxide oxygen atoms. In the case of the UOX:5-PIU complex, changes in the Raman spectra were too small for analysis (Figure S3). In order to define the vibrational modes responsible for the observed changes, we employed QM/MM methods at the MP2/6-31+G* level of theory (Figure 3 c). Calculations carried out for the organic species in their protein environment predict a band at 600 cm^−1^ (experimental 605 cm^−1^) for the 5-PMUA hydroperoxide (brown trace in Figure 3 c) and a band at 597 cm^−1^ (experimental 597 cm^−1^) for the MUA dianion (green). The former is of higher intensity as a result of a complex set of modes involving the stretching of the C5=Op1 bond and C5-Op1-Op2 bending coupled to ring distortions (Movie S1). Ring distortions account for the MUA 597 cm^−1^ band (Movie S2). The theory predicts a −9 cm^−1^ isotope shift (experimental −9 cm^−1^) for 5-PMUA featuring ^18^Op1 and ^18^Op2 peroxide oxygen atoms (orange trace). Overall, the calculations agree remarkably well with the experiment, identifying the 605 cm^−1^ 5-PMUA Raman band as a ‘signature’ for its peroxide state.

**Figure 3:**
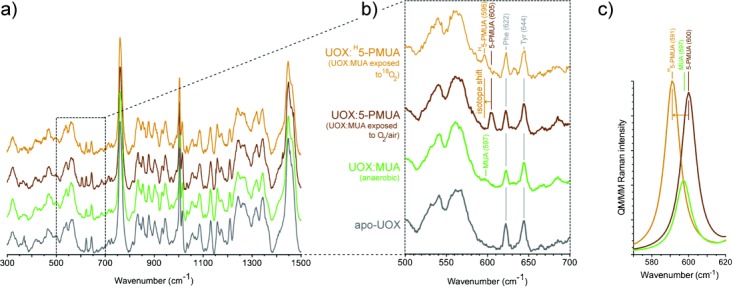
In crystallo Raman spectroscopic and QM/MM validation of MUA peroxidation. a) Raman spectra of apo-UOX (grey), anaerobic UOX:MUA (green), UOX:5-PMUA (brown), and UOX:^H^5-PMUA crystals (orange). ^H^5-PMUA (H for heavy) refers to MUA reacted with ^18^O_2_. b) Close-up of the 500–700 cm^−1^ spectral region. c) QM/MM Raman spectrum in the 570–620 cm^−1^ region for MUA dianion (green), 5-PMUA monoanion (brown), and ^H^5-PMUA monoanion (orange) at the MP2/6-31+G* level of theory. A band at 600 cm^−1^ (experimental 605 cm^−1^) is predicted for 5-PMUA. It is downshifted by −9 cm^−1^ (experimental −9 cm^−1^) in the presence of ^18^O peroxide oxygen atoms. A band at 597 cm^−1^ (experimental 597 cm^−1^) of lower intensity compared to the “peroxide signature band” is predicted for MUA.

Exposure to X-rays can lead to specific modifications in proteins and nucleic acids, including bond rupture.[9] In both UOX:5-PMUA and UOX:5-PIU crystals, we observed that the C5=Op1 bond is susceptible to selective radiolysis at low X-ray doses (Movie S3). We tracked peroxide radiolysis quantitatively using online Raman-assisted crystallography by performing multiple data collections interspersed by spectrophotometric measurements on a single UOX:5-PMUA crystal. In the case of UOX:5-PIU, only multiple X-ray data collections were performed. Details of the experimental protocol are given in Figures S4 and S5 in the Supporting Information. The resolution of all data sets is 1.3–1.4 Å with identical statistics (Table S1). The dose-dependent rupture of the C5=Op1 bond is accompanied by the loss of pyramidalization at C5, leading to a planar organic structure (Figures 4 a and S6 for 5-PMUA and 5-PIU, respectively). Concomitantly, a diatomic molecule of bond length shorter (1.2 Å) than the original peroxide Op1=Op2 bond is liberated and trapped above the planar structure. We interpreted the elongated electron density as molecular oxygen, because reactive oxygen species, such as superoxide anion or hydroperoxyl radicals, which are likely transiently formed in the process, are expected to rapidly convert to the more stable O_2_ molecule. Alternative assignments with a single oxygen atom produce difference density peaks, leaving no uncertainty on its diatomic nature (Figure S7). In crystallo UV/Vis absorption measurements are consistent with the formation of urate radical species upon peroxide radiolysis. Spectra collected on UOX:5-PIU crystals before and after X-ray exposure (Figure S8) show the development of broad features in the 310–550 nm region typical of urate radicals (hereafter Sub⋅).[10] These resonance-stabilized radicals are unreactive toward ground-state O_2_.[10] At a molecular level, the process of peroxide rupture is likely triggered by a one-electron reduction induced by the X-rays. Theoretical calculations confirm that one-electron reduction, unlike one-electron oxidation, leads to an unstable radical resulting in C5=Op1 bond break (Figure S9).

**Figure 4:**
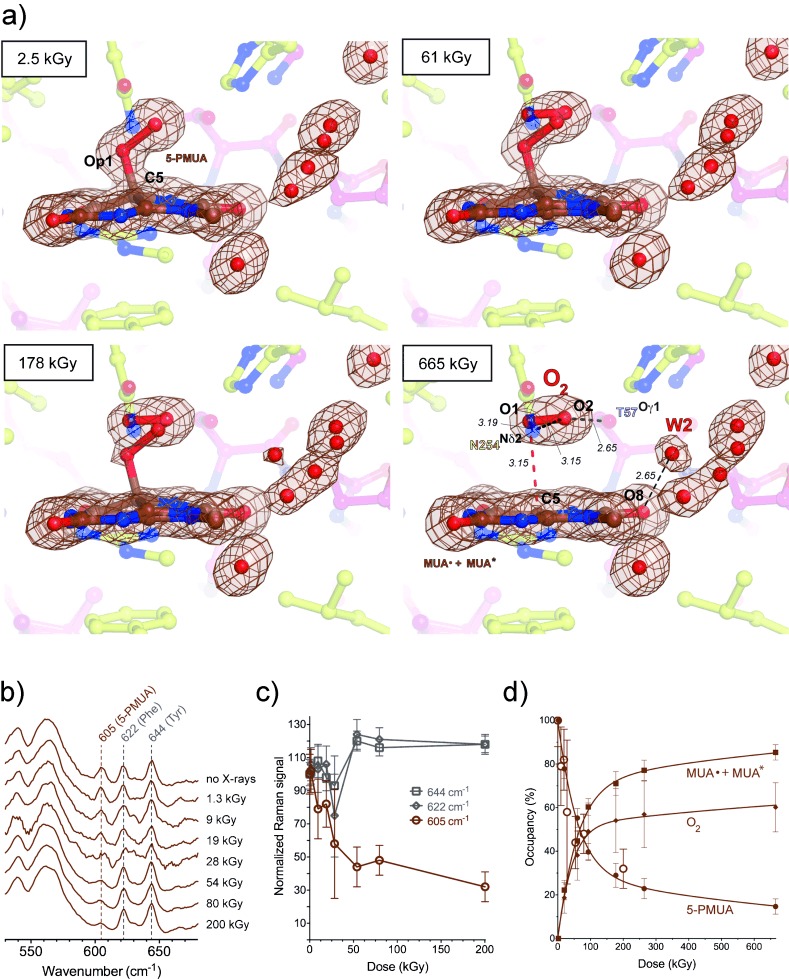
Peroxide radiolysis. a) Snapshots of UOX:5-PMUA at different X-ray doses. 2 *m* *F*_o_−*D* *F*_c_ electron density contoured at 1σ level is shown in brown for the organic moieties and solvent molecules in close proximity. Distances are in Å. Upon C5-Op1 rupture, dioxygen is trapped above the ensuing planar moiety.; b) In crystallo Raman spectroscopy shows a dose-dependent decrease of the 605 cm^−1^ 5-PMUA “fingerprint band” band; c) Decrease of Raman intensity is specific for the 605 cm^−1^ band. Two-tailed *p*-value analysis indicates a significant dose correlation for this band (p-value=0.0022), while the 622 cm^−1^ and 644 cm^−1^ bands assigned to Phe and Tyr, respectively,[17] are unaffected. Local scaling was carried out using the 565 cm^−1^ band. d) 5-PMUA decay is biphasic, thus indicating a mechanism of peroxide regeneration. 5-PMUA occupancies from crystallographic refinement are shown as circles. Open circles are 5-PMUA occupancies derived from the integration of the Raman band at 605 cm^−1^ (same as in panel c). O_2_ and (MUA^.^+MUA*, see Eq. (1) in the main text) occupancies are shown as diamonds and squares, respectively. Lines are the kinetic fit according to Eq. (1) of the main text. Kinetic constants are *k*_1_=11.28±0.36 MGy^−1^, *k*_2_=0.071±0.009 MGy^−1^ occupancy^−1^, *k*_3_=4.06±0.83 MGy^−1^, *k*_4_=0.12±0.03 MGy^−1^ occupancy^−1^, *k*_5_=1.56±0.37 MGy^−1^. See also Figure S10 for 5-PIU decay and its kinetic fit.

Dioxygen adopts a well-defined position and orientation above the flat organic molecule (Figures 4 a and S7). The O_2_ molecular axis lies parallel to the plane at a distance of 3.15 Å and is rotated by 15° compared to the peroxide bond. The O_2_ centroid is displaced by 0.85 Å from that of the peroxo group. Both O1 and O2 dioxygen atoms interact with N254(Nδ2) at distances of 3.19 Å and 3.09 Å, respectively, while the T57(Oγ1) atom is closest to O2 at 2.65 Å. The position of O_2_ observed here is consistent with that of room-temperature O_2_-pressurization experiments.[11] Peroxide breakdown also brings about a reorganization of the solvent around the organic moiety with the appearance of a solvent molecule (W2) bound to the O8 atom of the substrate. Raman analysis shows that peroxide rupture causes the 605 cm^−1^ ‘signature band’ to selectively decrease in a dose-dependent manner (Figure 4 b,c) consistent with our QM/MM calculations that assign this band to the stretching of the C5=Op1 bond.

The dose-dependent 5-PMUA occupancy from near-atomic resolution crystallographic refinement is in excellent agreement with its orthogonal estimation from the integration of the Raman signal at 605 cm^−1^ (Figure 4 d, filled and open circles). 5-PMUA decay follows a biphasic profile. 5-PIU decay displays the same behavior (Figure S10). A biphasic curve is not consistent with a simple (Peroxide→Sub⋅+O_2_) decomposition process. Such a mechanism should result in a monoexponential decay to zero, as observed for the irreversible debromination of 8-bromo-2′-deoxyguanosine in modified DNA.[12] This observed decay can be rationalized assuming a pathway of peroxide regeneration acting alongside its decomposition. A mechanism of X-ray induced repair has recently been proposed for -S-S- bridges.[13] A satisfactory kinetic fit (log-goodness-of-fit=1.33, Figure 3 d, see also Figure S10 for 5-PIU kinetic fit) was obtained assuming the following working scheme [Eq. (1)]:

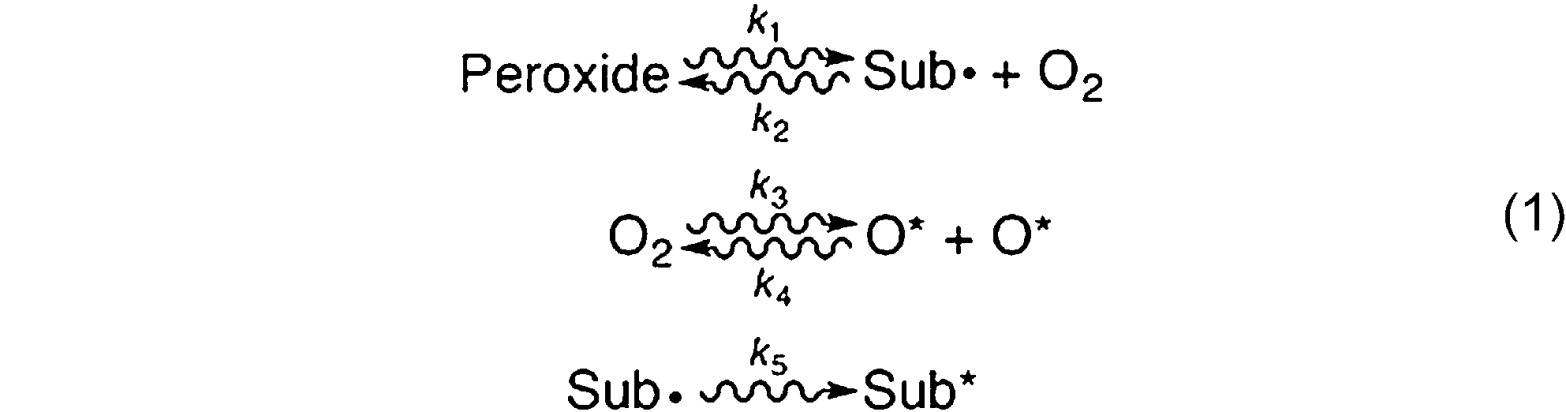


It accounts for a recombination reaction (described by *k*_2_) between Sub⋅ and superoxide, promoted by the one-electron reduction of O_2_ by solvated electrons.[9a] Superoxide is readily formed by ionizing radiation in the presence of dioxygen.[14] O* refers to O_2_ (or H_2_O) decay species (for example OH⋅), while Sub* takes into account a nonreactive form of the substrate (for example the one-electron oxidation product of Sub⋅). Sub* and Sub⋅ are crystallographically indistinguishable. We find that 5-PMUA is more susceptible to radiolysis than 5-PIU (*k*_1_=11.29±0.36 and 6.94±0.32 MGy^−1^ for 5-PMUA and 5-PIU, respectively), possibly as a result of an inductive effect of the methyl group. The rate constant *k*_2_ is independent of the specific substrate (0.071±0.009 and 0.073±0.010 MGy^−1^ occupancy^−1^ for MUA⋅ and UA⋅, respectively), thus suggesting an equal probability to undergo the one-electron reduction required to kick-start radical recombination.

X-ray crystallography at near-atomic resolution together with in crystallo Raman spectroscopy and QM/MM methods offer unambiguous evidence for a C5-peroxide intermediate in cofactor-free UOX catalysis. Peroxide radiolysis followed by online Raman-assisted X-ray crystallography afforded exquisite insight into the elusive configuration of the reactants, leading to the peroxo intermediate by a radical recombination mechanism.
