# Supplementary figures and images for: Direct Evidence for a Peroxide Intermediate and a Reactive Enzyme–Substrate–Dioxygen Configuration in a Cofactor-free Oxidase
Source: Angew Chem Int Ed Engl. 2014 Oct 14;53(50):13710–4. doi: 10.1002/anie.201405485 (PMC4502973; doi:10.1002/anie.201405485)

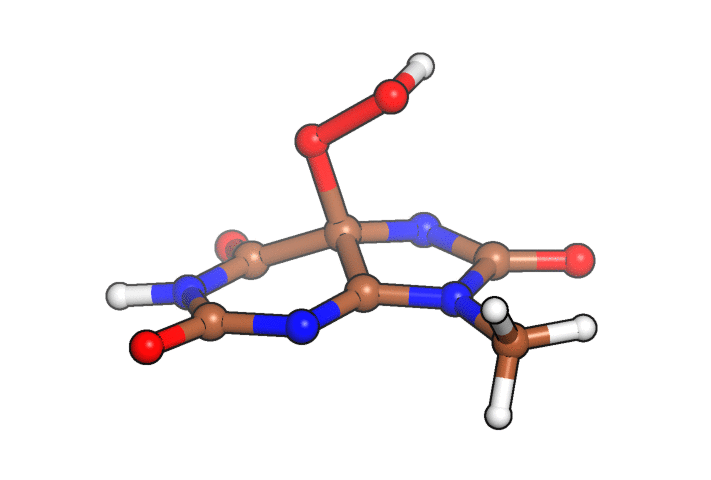

Supplement: Supplementary file 1 [file anie0053-13710-sd1.gif]

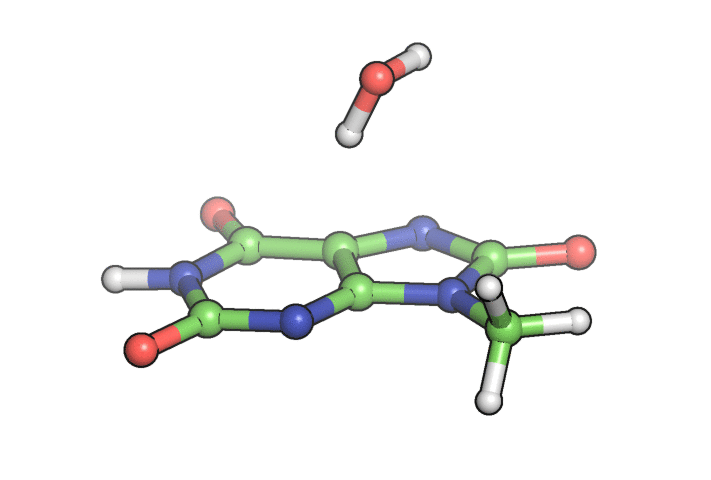

Supplement: Supplementary file 2 [file anie0053-13710-sd2.gif]

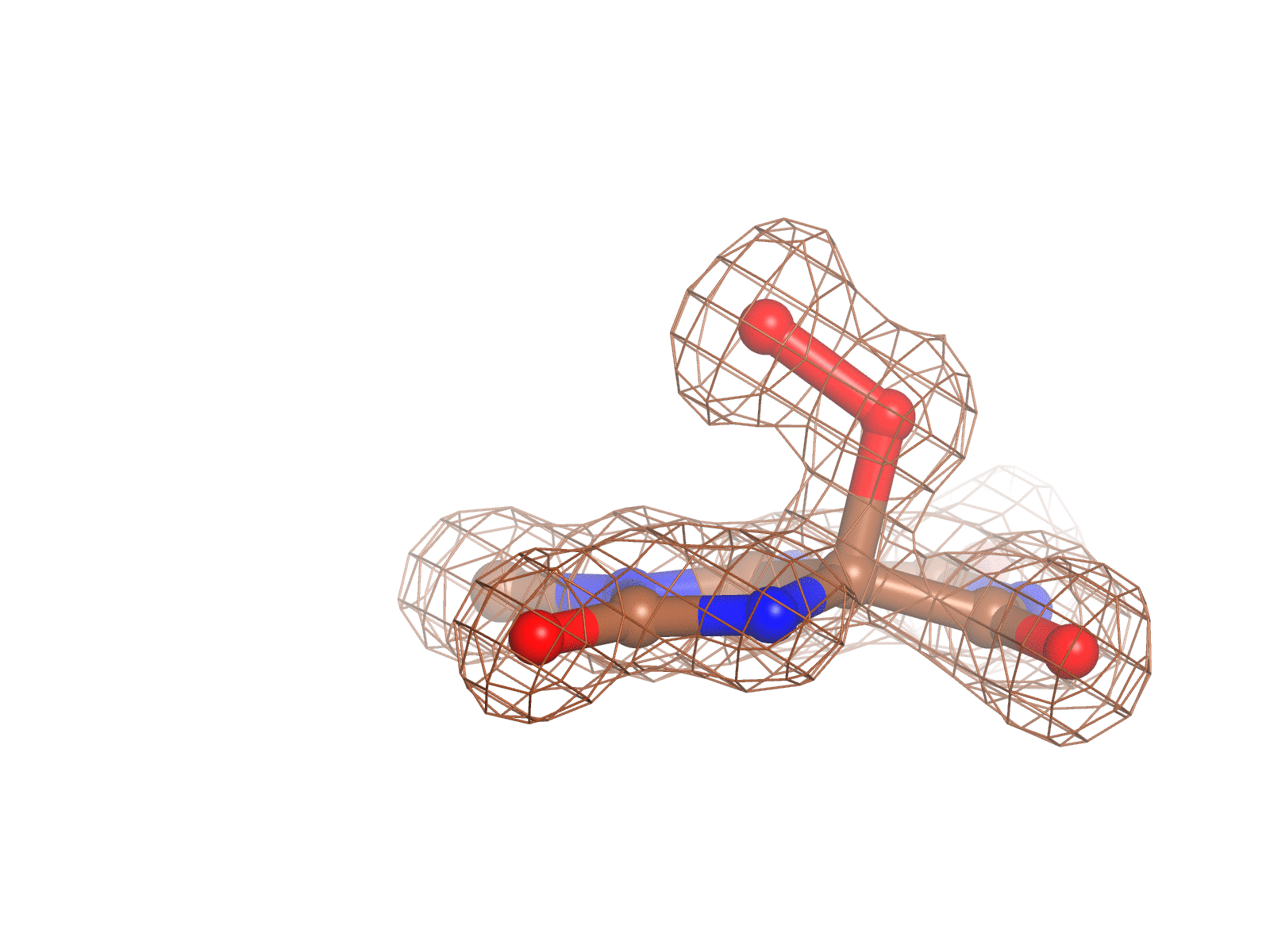

Supplement: Supplementary file 3 [file anie0053-13710-sd3.gif]
