# Supplementary material for: Direct Evidence for a Peroxide Intermediate and a Reactive Enzyme–Substrate–Dioxygen Configuration in a Cofactor-free Oxidase
Source: Angew Chem Int Ed Engl. 2014 Oct 14;53(50):13710–4. doi: 10.1002/anie.201405485 (PMC4502973; doi:10.1002/anie.201405485)
Supplement: Supplementary file 4 [file anie0053-13710-sd4.pdf]

Angewandte Chemie
Eine Zeitschrift der Gesellschaft Deutscher Chemiker

Supporting Information
© Wiley-VCH 2014
69451 Weinheim, Germany

Direct Evidence for a Peroxide Intermediate and a Reactive Enzyme–Substrate–Dioxygen Configuration in a Cofactor-free Oxidase**

*Soi Bui, David von Stetten, Pablo G. Jambrina, Thierry Prangé, Nathalie Colloc'h, Daniele de Sanctis, Antoine Royant, Edina Rosta, and Roberto A. Steiner**

anie_201405485_sm_miscellaneous_information.pdf
anie_201405485_sm_Bui_Movie_S1.gif
anie_201405485_sm_Bui_Movie_S2.gif
anie_201405485_sm_Bui_Movie_S3.gif

Supplementary text

UOX expression and purification

Two *Aspergillus flavus* UOX preparations were used in this study. Purified UOX expressed in *Saccharomyces cerevisiae* and commercialized under the brand name Fasturtec was a kind gift from Sanofi-Aventis. Alternatively, untagged UOX was expressed in *E. coli* using a codon-optimised synthetic cDNA (Genscript, USA) inserted into a pET24b vector (Novagen). Protein expression was performed in *E.coli* BL21(DE3) cells induced at OD_{600} ~0.6-0.8 with 0.2 mM IPTG at 20°C for ~20 hours. Pelleted cells were re-suspended in 50 mM Tris-HCl (pH 8.0), 250 mM NaCl, supplemented with lysozyme, DNAse and a protease inhibitor cocktail and lysed with sonication. Protein purification was performed using a combination of ammonium sulphate precipitation, DEAE and Resource Q ion exchange, Phenyl Sepharose hydrophobic interaction, and Superdex 75 size-exclusion chromatographic steps. Fractions were analysed by SDS-PAGE and the purest fractions pooled for further work.

Crystallization

UOX was exchanged against a Tris-HCl or Tris-acetate buffer (50 mM, pH 8.0) and concentrated to 20 mg/ml. Anaerobic crystals of the complexes were obtained at room temperature using N_2-purged UOX solutions saturated with either 9-methyl uric acid (MUA) or uric acid (UA) at pH 8.0 in 50 mM Tris-HCl (or Tris-acetate buffer) and 8% PEG 8000 as crystallizing agent. The batch method was used throughout by mixing reservoir and protein solutions in a 2:1 ratio. UOX crystals typically reach dimensions of 400×400×400 μm^3.

For cryoprotection, crystals were transferred to a reservoir solution enriched with 20% MPD for five minutes and then rapidly quenched and stored in liquid nitrogen. All oxygen-free crystal manipulations were carried out in an anaerobic chamber (Belle Technology) equipped with a stereomicroscope at an O_2 pressure lower than 2 ppm. Crystals for the UOX:5-PMUA peroxide complex were obtained by transferring anaerobic UOX-MUA crystals to normoxic conditions, i.e. outside the glove box, prior to cryoprotection. Aerobic exposure at atmospheric pressure for approximately 60 minutes results in the formation of 5-PMUA with full occupancy. The use of an in-house developed O_2 pressurization device allows formation of the peroxide in much shorter times. Crystals of ^{heavy}5-PMUA were prepared in the glove box by incubating for about 12 hrs anaerobically-grown UOX:MUA crystals in a $^{18}O_2$-containing reservoir solution. The latter was prepared by flushing 75 µl of anaerobic reservoir with 50 ml of 98% $^{18}O_2$ (CK Isotopes, Ibstock, Leicestershire, UK) in a 7 ml bijoux. Crystals for the UOX:5-HIU peroxide complex were obtained by soaking UOX:UA crystals in the cryoprotectant solution further enriched with 10 mM H_2O_2 under anaerobic conditions.

X-ray data collection

Data collections on UOX crystals belonging to space group *I*222 were carried out either using our in-house sealed-tube X-ray instrument (Agilent Nova) or synchrotron radiation (ESRF, Grenoble-France and Diamond Light Source, Didcot-U.K.). For synchrotron measurements X-ray dose calculations were performed with the program RADDOSE-3D [1] to take into account the larger crystal dimensions compared to the X-ray beam cross-section. The

standard protocol for multiple data collections involved a series of identical acquisitions with a rotation range of 180° at low dose (data sets) interspersed by 'burn' acquisitions. Data collection parameters and statistics are given in Table S1. None of the general global features that typically accompany X-ray radiation damage effect such as increase of cell dimensions, increased overall ADP values are present here as the X-ray dose provided is about 40-100 lower than the Garman limit.[2] Typical specific damages in proteins are also rupture of disulfide bridges and decarboxylation of acidic residues.[3] Whilst there are no disulfide bridges in UOX, no sign of Asp/Glu decarboxylation are observed.

Crystallographic refinement

The programs *COOT*[4] and *Refmac*5[5] were used throughout for model rebuilding and anisotropic refinement with riding hydrogen atoms, respectively. Refinement statistics are given in Table S1. Refinement was additionally carried out with *phenix.refine*[6] and *Shelxl*[7] software packages for cross-validation of occupancy (q) values. Isomorphous difference maps were calculated within *Phenix* [8].

For occupancy refinement, the sum $q_{PEROXIDE}$ + q_{FLAT} (FLAT refers to the planar radical moieties (Sub• + Sub*) arising from peroxide rupture) was constrained to unity whereas $q_{DIOXYGEN}$ was refined independently. The $q_{PEROXIDE}$ + q_{FLAT} = 1.0 condition reflects the observation that geometrically invariant atoms in the PEROXIDE → FLAT reaction are present at full occupancy whereas electron density for the diatomic specie is lower. In test refinement runs we also removed the $q_{PEROXIDE}$ + q_{FLAT} = 1.0 constrain. This

resulted in occupancy values, whose sum was essentially unitary (0.97-0.99). We therefore considered justified to keep this constrain throughout.

Kinetic fitting

Several reaction schemes were tested by performing kinetic fitting of dose-dependent occupancies with the package Dynafit.[9] The reaction scheme below provided the best results assessed by fitting quality (log(goodness of fit)) and lowest errors on the kinetic constants derived (errors lower than 35%)

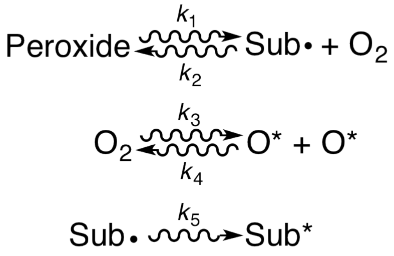

Changes in concentrations of the chemical species were computed by solving an initial value problem described by the following system of differential equations:

$$\frac{d[\text{peroxide}]}{d\text{D}} = -k_1[\text{peroxide}] + k_2[\text{Sub}\,\cdot][\text{O}_2]$$

$$\frac{d[\text{Sub}\,\cdot]}{d\text{D}} = +k_1[\text{peroxide}] - k_2[\text{Sub}\,\cdot][\text{O}_2] - k_5[\text{Sub}\,\cdot]$$

$$\frac{d[\text{O}_2]}{d\text{D}} = +k_1[\text{peroxide}] - k_2[\text{Sub}\,\cdot][\text{O}_2] - k_3[\text{O}_2] + k_4[\text{O}^*][\text{O}^*]$$

$$\frac{d[\text{O}^*]}{d\text{D}} = +k_3[\text{O}_2] + k_3[\text{O}_2] - k_4[\text{O}^*][\text{O}^*] - k_4[\text{O}^*][\text{O}^*]$$

$$\frac{d[\text{Sub}^*]}{d\text{D}} = +k_5[\text{Sub}^*]$$

with conditions

$$[\text{peroxide}]_{\text{D}=0} = 100$$

$$[O_2]_{D=0} = [O^*]_{D=0} = [Sub\cdot]_{D=0} = [Sub^*]_{D=0} = 0$$

We emphasize that although Scheme 1 allows a satisfactory kinetic fit of the data it contains speculative elements and at present should be considered as a working model.

Raman measurements

Non-resonant Raman spectra were recorded at 100 K at the ESRF (Grenoble) either off-line at ID29S-Cryobench laboratory or on-line at the ID29 beamline using an inVia Raman microspectrometer (Renishaw, Gloucestershire, UK) equipped with a near-IR 785 nm diode laser.[10] For on-line measurements both the spectrometer and the laser located at the Cryobench were connected via a 50 m-long optical fibre cable to the Raman head installed at the beamline and driven by a precise motorized device. The Raman laser was co-axial (antiparallel) with the X-ray beam. This experimental procedure allows a single crystal to be alternatively probed by X-ray crystallography and Raman spectroscopy in an interleaved manner without any manual intervention on the sample. The same location of the crystal (within 10 μm) was probed by microspectrometry using a laser power of 50 mW at the sample position and 2000 s accumulated exposure time for each spectrum. As crystal orientation is known to strongly affect the relative band intensities in Raman spectra, e.g. due to polarization effects, the same spindle axis position was used for all Raman measurements. Non-resonant Raman data acquisition with laser irradiation at 785 nm does not result in detectable degradation of the structural and spectroscopic signatures of the samples. To obtain peak intensities, a curve-fitting procedure was applied

using a group of Lorentzian peaks between 500 cm^{-1} and 700 cm^{-1} with a linear baseline.

UV-visible light absorption microspectrophotometry

UV-visible light absorption spectra of non-irradiated and irradiated crystals were obtained at the *in crystallo* spectroscopy facility ID29S Cryobench at the ESRF, Grenoble, France. The reference white light is provided by a balanced deuterium-halogen lamp (Mikropack DH2000-BAL, Ocean Optics). Spectra are recorded using a fixed-grating spectrophotometer with a CCD detector (QE65Pro, Ocean Optics).

Theoretical calculations

MD simulations were initiated based on the available crystal structures. The starting structures were prepared using the CHARMM[11] software and the MD simulations were performed with NAMD[12] (version 2.9). The coordinates of the hydrogen atoms were generated with CHARMM using the standard protonation states for the titratable residues. As we lack experimental information about the protonation and the total charge of the substrate, we identified and tested several valence models. The positions of the hydrogen atoms of the ligand were optimized in the absence of solvent at the B3LYP/6-31+G* level of theory using QCHEM[13] and the atomic charges in the substrate were calculated using the CHELPG[14] algorithm. The protein was placed in the center of a cubic box that extends at least 10 Å in all directions from the system. This cube was filled with water molecules and the total charge of the system was neutralized using either K^+ or Cl^- ions. The Particle Mesh Ewald method was used to treat the electrostatics of the periodic

boundary conditions with a non-bonded cutoff of 13.5 Å and with an electrostatic force shifting function and a van der Waals switching function between 10 and 12 Å. The CHARMM27 force field was used throughout the simulations. The protein and the solvent were energy minimized following a two-step procedure, while the substrate was kept fixed at its crystallographic coordinates. Firstly, the non-backbone atoms and non-crystallographic water molecules were minimized for 9000 steps, subsequently all the atoms were minimized for 20000 steps. Finally, the system was equilibrated for 3 ns imposing harmonic restraints on the backbone atoms and oxygens of the crystallographic waters with the substrate fixed. Langevin dynamics was run at a temperature of 298 K with a 1 fs time step and a damping coefficient of 1 ps^{-1}. Once the system was equilibrated, we carried out QM/MM minimizations with the MUA and the O_2 in the QM region at the B3LYP/6-31+G* and the MP2/6-31+G* levels of theory for 100 steps. The Raman spectra of the ligands were calculated in the protein environment by determining the vibrational frequencies of the QM region. We used the sum of Lorentzian functions located at every fundamental vibrational frequency, normalized based on the calculated intensities, and using a FWHM of 10 cm^{-1}. We tested the geometric effects on the substrate in the QM region of adding or removing a single electron and carried out QM/MM minimizations as explained above. After the minimizations with an added electron, the HOMO and HOMO-1 orbitals of the system and the CHELPG atomic charges were calculated using Gaussian 09.[15]

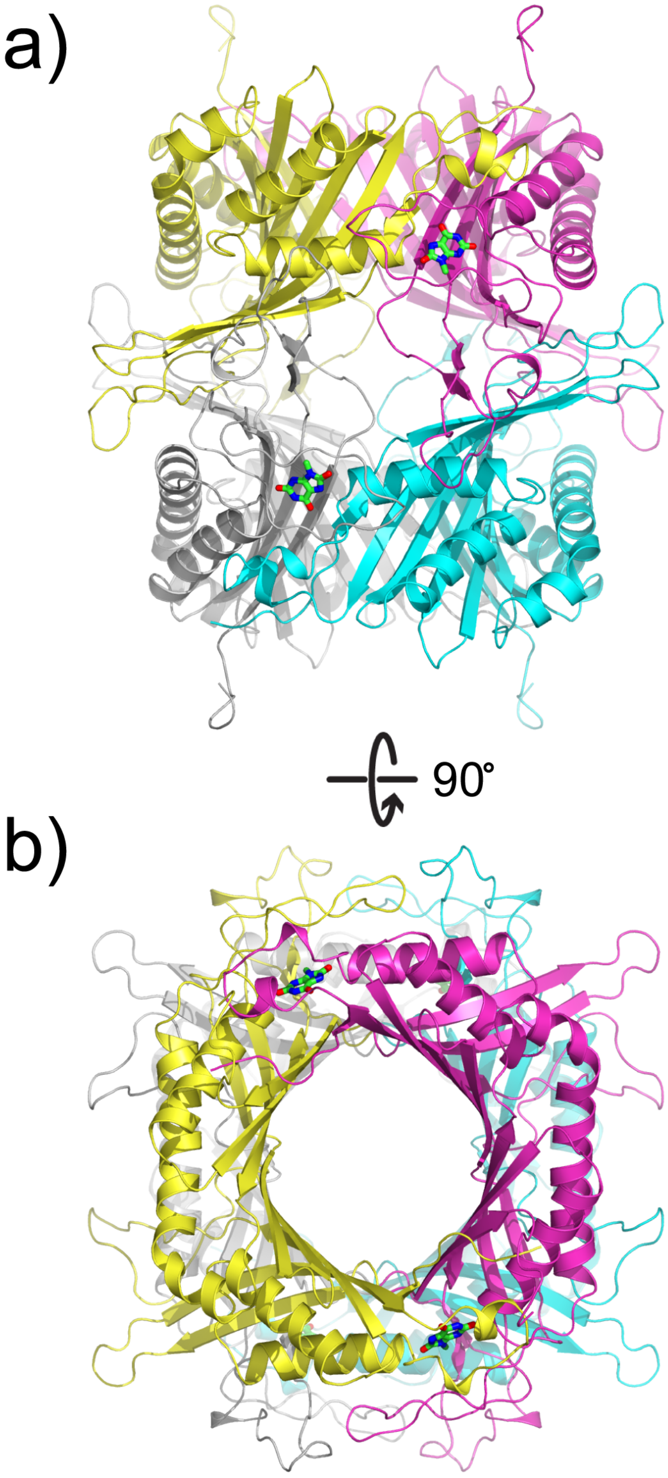

Figure S1. UOX tetramer. Side- (**A**) and top-view (**B**) of the UOX tetramer of 222 point symmetry. It crystallizes in space group *I*222 with one monomer in the a.u. UOX subunits A, B, C, D are represented as cartoons and colored in yellow, magenta, grey and cyan, respectively. The tetramer binds four molecules of substrate (shown in green as ball-and-stick) in each active site located at the interface between two monomers.

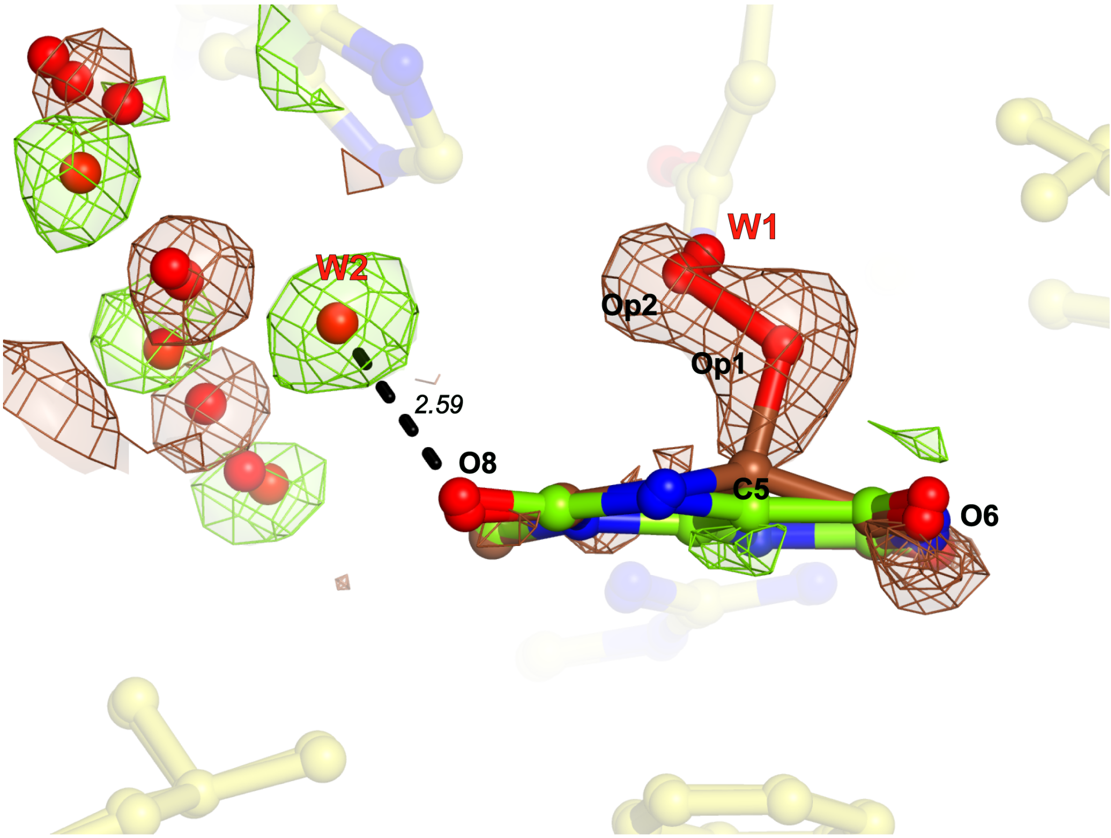

Figure S2. MUA→5-PMUA transition. $(F_{o(UOX:5\text{-}PMUA)}\text{-}F_{o(UOX:MUA)})\exp(i\varphi_{(UOX:5\text{-}PMUA)})$ isomorphous difference map contoured at the +4.5σ and -4.5σ levels in brown and green, respectively. The view is from the C5-side of the organic molecules, roughly rotated by 180° compared to Figure 2 of the main text. The isomorphous difference map highlights the extra density for the C5-Op1 and Op1-Op2 bonds as well as the geometry changes induced by the formation of the peroxide. The C5 atom shifts above the MUA plane by 0.4 Å whilst the O6 and O8 atoms move below by 0.3 Å and 0.2 Å, respectively. Peroxidation alters the solvent network. In particular, the W2 solvent molecule bound in the anaerobic E•S complex to the O8 atom moves away from in the E•peroxide complex.

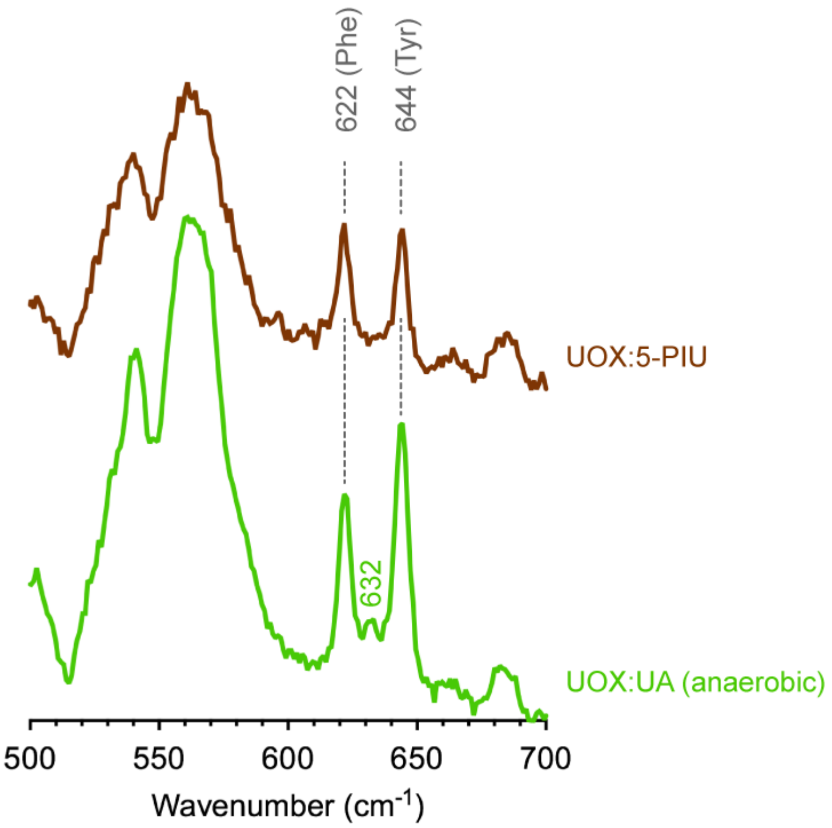

Figure S3. Off-line *in-crystallo* Raman spectra of the UOX:5-PIU (brown line) and anaerobic UOX:UA (green line) complexes. Differently from MUA, peroxidation of UA, confirmed by X-ray crystallography, does not provide a robust Raman signature under the conditions employed. Only minor differences are observed in the 600 cm^{-1} region. These, however, are too small to allow a reliable analysis.

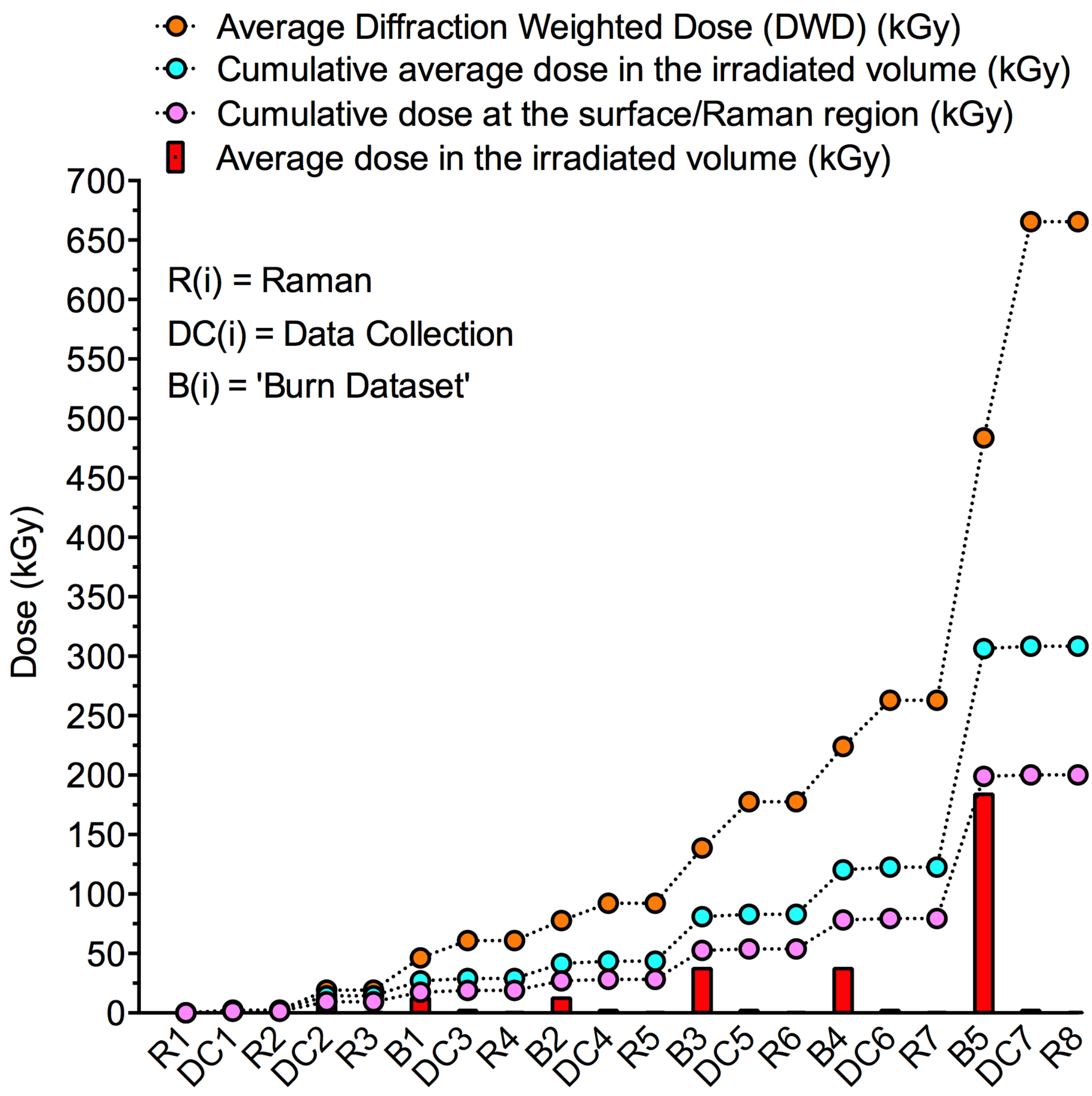

Figure S4. Protocol employed for the combined crystallographic/online Raman measurement on UOX:5-PMUA performed at the ID29 beamline (ESRF, Grenoble). A total of seven data collections, DC(i), interspersed by 'burn datasets', B(i), and online Raman microspectrophotometry measurements, R(i), were acquired from a single UOX:5-PMUA crystal. Dose calculations were performed using the program RADDOSE-3D (www.raddo.se) (*20*) to take into account the bigger crystal size compared to the X-ray beam dimensions The diffraction weighted dose (DWD) represents the effective average dose that is observed in the diffraction pattern [16]. Raman microspectrophotometry probes the surface of the irradiated crystal volume. The dose at the surface considering the experimental geometry was calculated from the RADDOSE-3D output:
Average_dose_surface = average_dose_irradiated_volume/1.54.

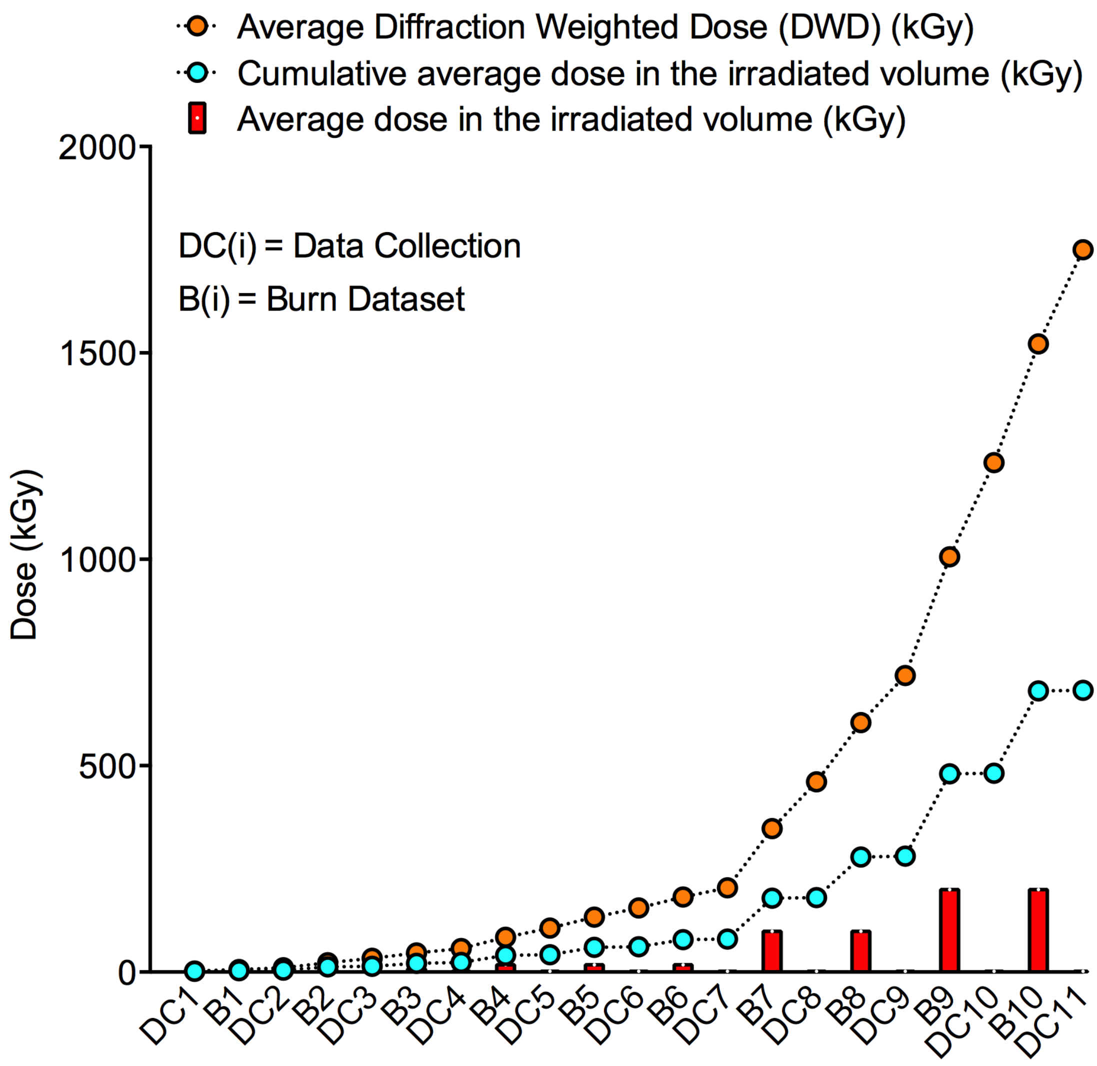

Figure S5. Protocol employed for the crystallographic measurement performed on UOX:5-PIU at the I02 beamline (DLS, Dicot). A total of eleven data collections, DC(i) interspersed by 'burn datasets', B(i), were acquired from a single UOX:5-PIU crystal. Dose calculations were performed using the program RADDOSE-3D (www.raddo.se) (*20*) to take into account the bigger crystal size compared to the X-ray beam dimensions The diffraction weighted dose (DWD) represents the effective average dose that is observed in the diffraction pattern [16]. Raman microspectrophotometry was not performed on this sample due to the low intensity of 5-PIU specific bands (Figure S3).

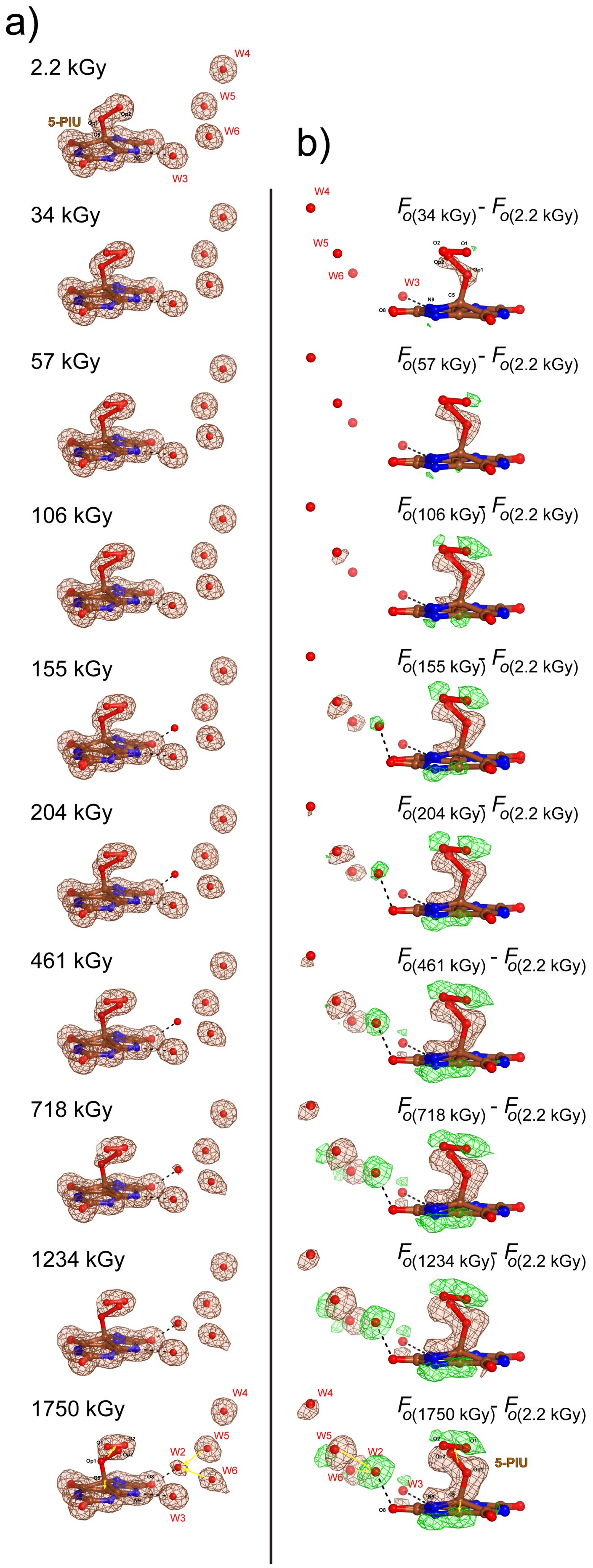
a)
2.2 kGy
5-PIU
W4
W5
W6
W3
34 kGy
57 kGy
106 kGy
155 kGy
204 kGy
461 kGy
718 kGy
1234 kGy
1750 kGy
W2
b)
F o(34 kGy) - F o(2.2 kGy)
F o(57 kGy) - F o(2.2 kGy)
F o(106 kGy) - F o(2.2 kGy)
F o(155 kGy) - F o(2.2 kGy)
F o(204 kGy) - F o(2.2 kGy)
F o(461 kGy) - F o(2.2 kGy)
F o(718 kGy) - F o(2.2 kGy)
F o(1234 kGy) - F o(2.2 kGy)
F o(1750 kGy) - F o(2.2 kGy)

Figure S6. Dose-dependent rupture of the C5-Op1 bond of 5-PIU. (**A**) Data sets for UOX:5-PIU were collected on the same crystal at increasing X-ray dose according to the protocol in Figure S5. Upon X-ray exposure the C5-Op1 bond breaks resulting in the accumulation of O_2 and the planar UA species (UA• + UA*, see Scheme 1 in the main text). The (W2) water molecule moves close to the O8 atom at H-bond distance. Organic moieties and O_2 and are shown as ball-and-stick models. Waters in close proximity are shown as spheres. Carbon, oxygen, and nitrogen atoms are shown in brown, red and blue, respectively. $2mF_o$-DF_c electron density maps at the 1.0σ level are shown in brown. Hydrogen bonds are shown as dashed black lines; (**B**) ($F_{o(x\ kGy)}$-$F_{o(2.2\ kGy)}$)exp($i\varphi_{(2.2\ kGy)}$) isomorphous difference maps contoured at the +5.5σ (green) and -5.5σ (brown) levels; They highlight the bond breaking process with the concomitant flattening of the residual organic specie and trapping of O_2 as well as the reorganization of the solvent network. The solvent molecule W2 is mostly formed as a result of the partial relocation of W5 and W6.

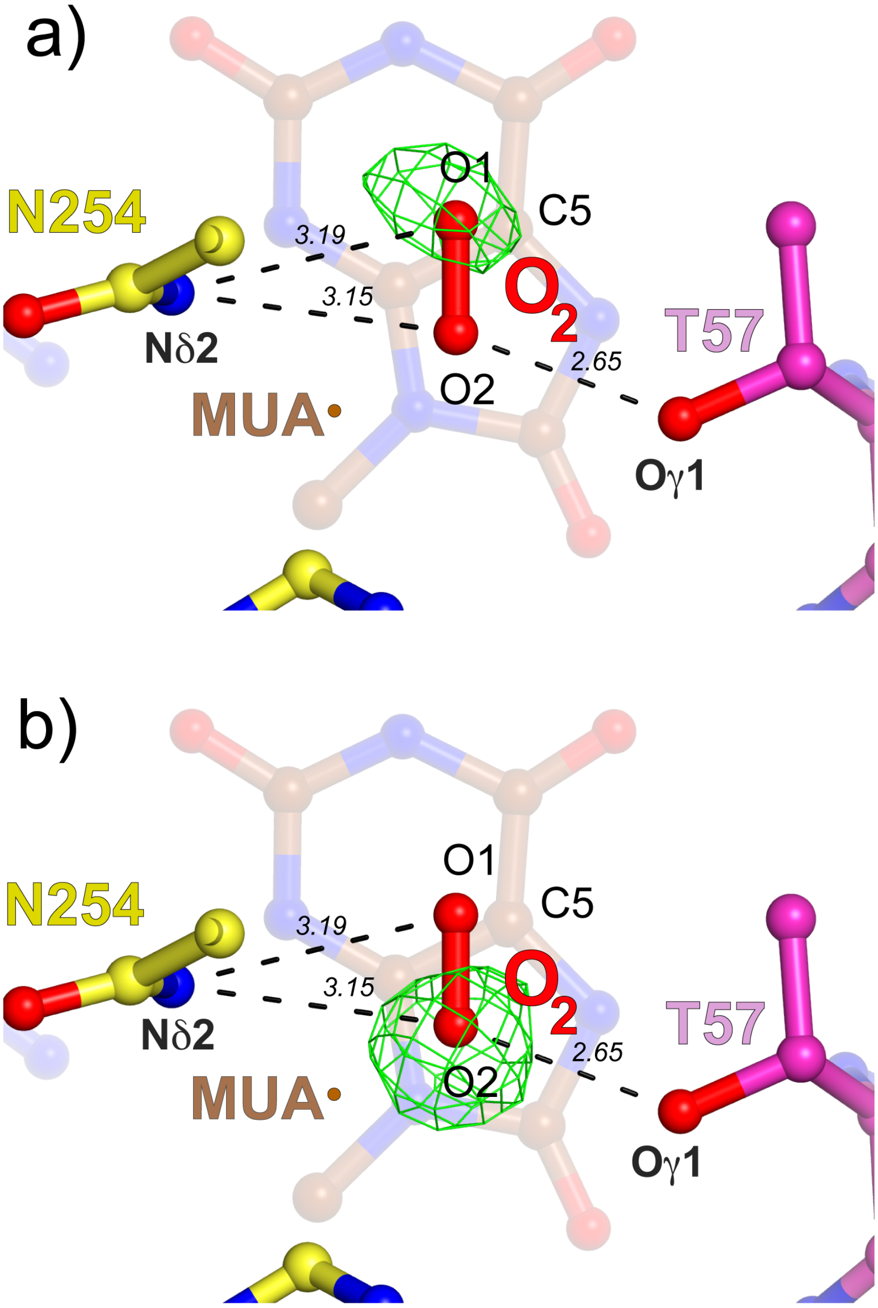

Figure S7. Top-view (O_2-view) of the UOX active site in the presence of photolyzed 5-PMUA (dose = 288 kGy). O1 (panel A) and O2 (panel B) atoms of molecular oxygen (shown as red ball-and-stick representation) have been selectively removed from crystallographic refinement and the resulting difference mF_o-DF_c electron density map at the 1.34 Å resolution is shown in green at the 4σ level. It highlights the diatomic nature of the molecule liberated upon rupture of the C5-Op1 bond. Carbon atoms of selected residues from different UOX protomers are shown in yellow and magenta, respectively. Carbons of the MUA radical are shown in brown. Oxygen and nitrogen atoms are in red and blue, respectively. Broken black lines indicate H-bonds between dioxygen and UOX residues. Distances are in Å.

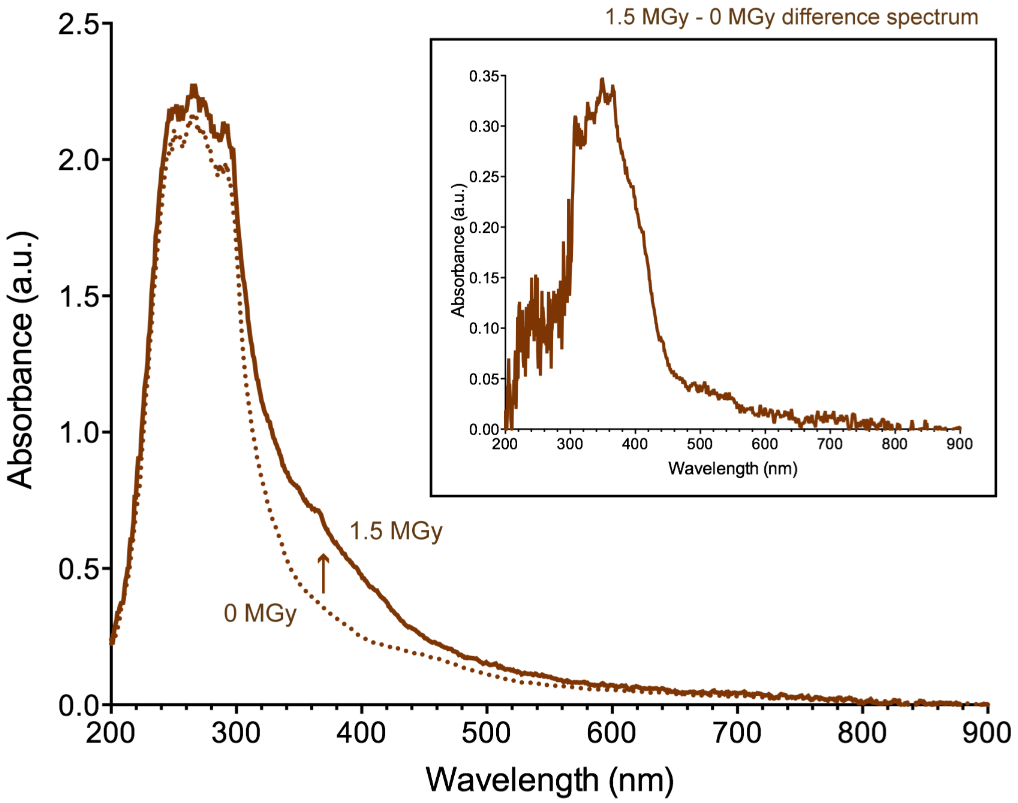

Figure S8. UV/Vis changes upon exposure of UOX:5-PIU crystals to X-rays**.** The spectrum before X-ray exposure is shown in brown as a dotted line. The spectrum after X-ray exposure (1.5 MGy) is represented by the continuous line. The inset shows the difference spectrum. Following X-ray exposure there is an increase in absorbance in the broad 310-550 nm region exhibiting a maximum at around 360-380 nm. This is consistent with the formation of a resonance-stabilized urate radical species.[17]

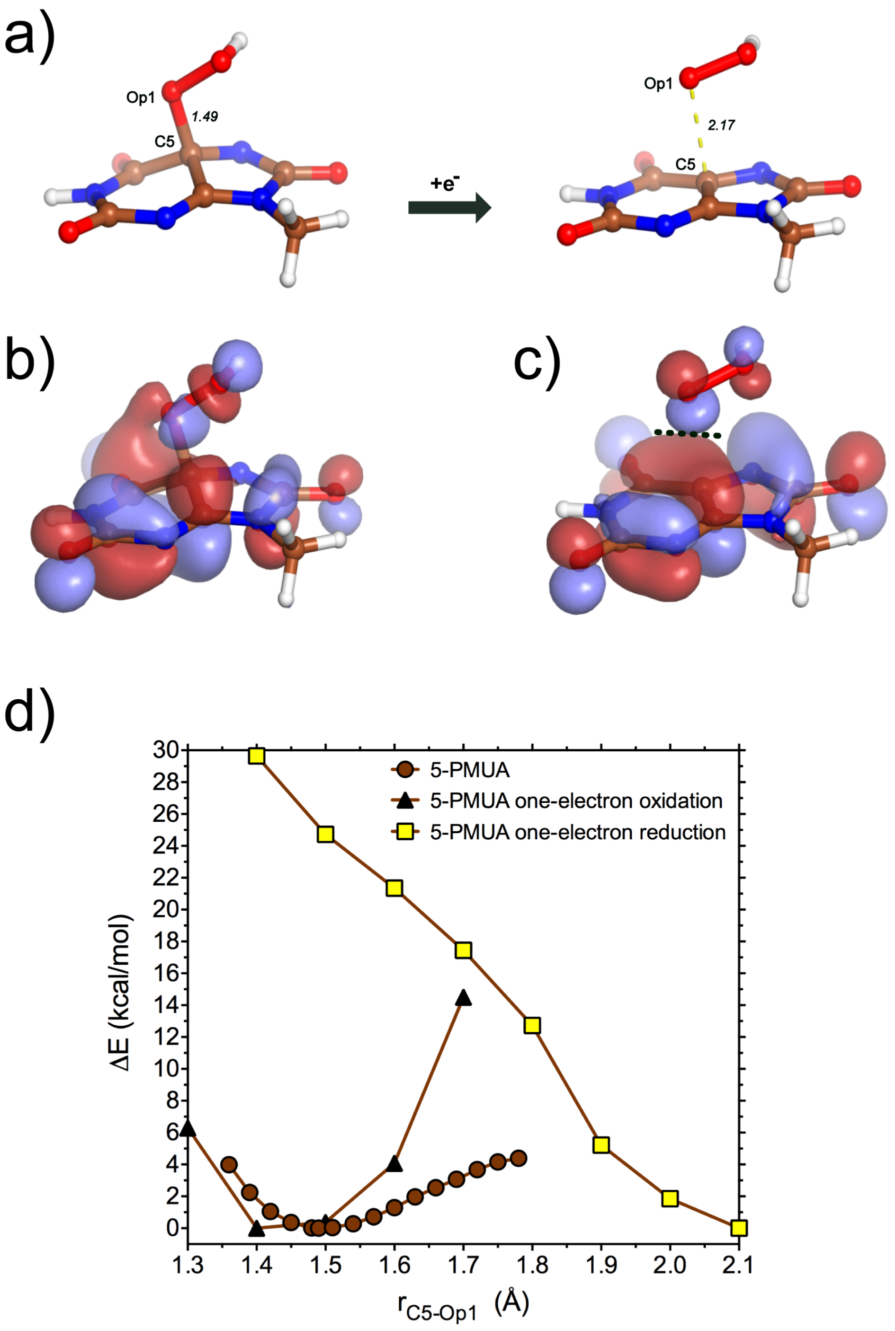

Figure S9. One-electron reduction of 5-PMUA results in rupture of the C5-Op1 bond. (**A**) QM/M calculations at the MP2/6-31+G* level of theory show that one-electron reduction destabilizes the 5-PMUA hydroperoxide leading to C5-Op1 bond break. Carbon, nitrogen, oxygen and hydrogen atoms are shown in brown, blue, red, and white, respectively. Distances are in Å; (**B,C**) Upon reduction the extra electron is injected into the LUMO orbital of 5-PMUA (**B**). The node (black dashed line in **C**) along the C5-Op1 bond is more clearly visible once the peroxide has dissociated as seen from the HOMO orbital of reduced 5-PMUA (**C**); (**D**) Energy profile of 5-PMUA minimized within UOX as a function of the C5-Op1 distance. For 5-PMUA (circles) the minimum is found at a C5-Op1 bond distance of 1.49 Å. The removal of one electron (triangles) results in the shortening (1.40 Å) of the optimal C5-Op1 bond distance. On the other hand, addition of one electron (squares) shifts this minimum to significantly larger distances consistent with bond rupture.

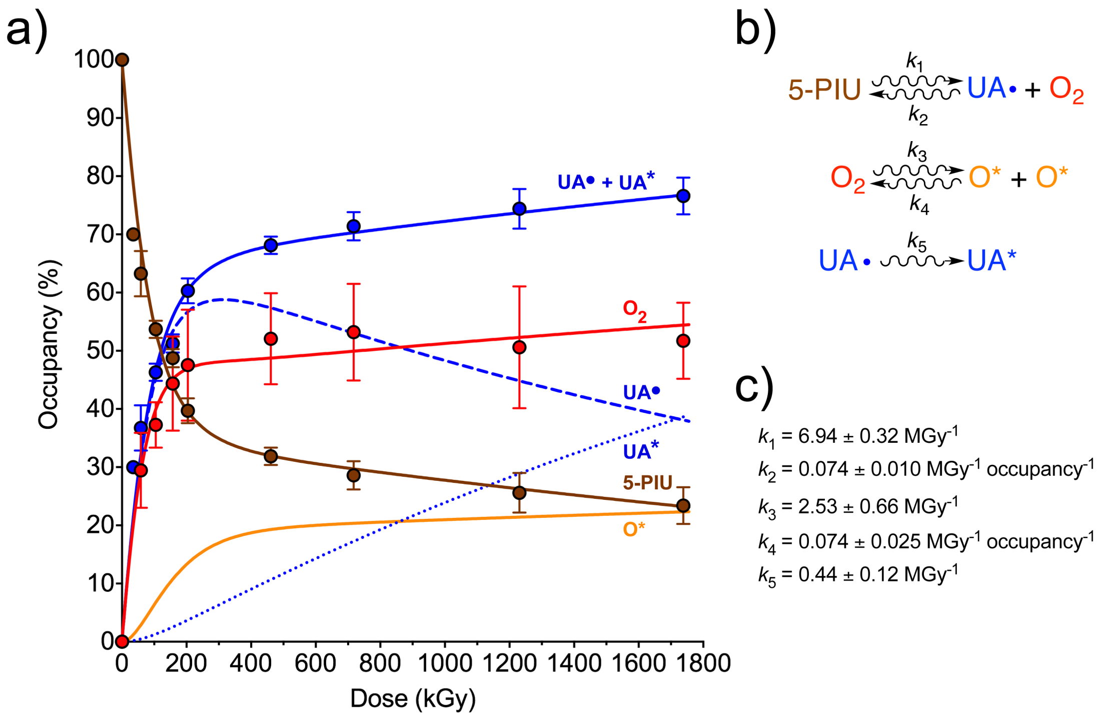

Figure S10. Dose-dependent 5-PIU rupture and regeneration**.** **(**A**)** Occupancies from crystallographic refinement (brown (5-PIU), red (O_2) and blue circles (UA* +UA•)) were fit to the reaction scheme in panel B using the package Dynafit.[9] Lines represent the dose-dependent occupancies of the different species calculated from the five kinetic constants derived from the fitting procedure; (B) Reaction scheme. The scheme accounts for the recombination reaction between UA• (one-electron oxidation product of UA dianion) and superoxide promoted by the one-electron reduction of the UA•-O2 complex by radiolytically-produced solvated electrons. O* refers to any O_2 decay species (for example OH•) whilst UA* indicates a non-reactive form of the substrate (for example the one-electron oxidation product of Sub•). UA* and UA• are crystallographically indistinguishable. (C) Dose-dependent kinetic constants obtained from the least-squares fitting procedure.

Table S1. Summary of X-ray data collection and refinement statistics.

Data set	UOX:MUA Anaerobic	UOX:5-PMUA DC1	UOX:5-PMUA DC2	UOX:5-PMUA DC3	UOX:5-PMUA DC4	UOX:5-PMUA DC5	UOX:5-PMUA DC6	UOX:5-PMUA DC7
Source	BM30/ESRF	ID29/ESRF	ID29/ESRF	ID29/ESRF	ID29/ESRF	ID29/ESRF	ID29/ESRF	ID29/ESRF
Crystal size (μm^3)	-	450x450x450	450x450x450	450x450x450	450x450x450	450x450x450	450x450x450	450x450x450
Beam FWHM, vxh (μm^2)	-	30x70	30x70	30x70	30x70	30x70	30x70	30x70
Flux (photons/s)*	-	1.36E10	1.36E10	1.36E10	1.36E10	1.36E10	1.36E10	1.36E10
Total exposure time (s)	-	14.4	14.4	14.4	14.4	14.4	14.4	14.4
Diffraction weighted dose (kGy)	-	2.5	19	60	92	178	263	665
Wavelength (Å)	1.18200	0.97625	0.97625	0.97625	0.97625	0.97625	0.97625	0.97625
Resolution range (Å)<br>Highest res. Bin (Å)	30.54-1.50<br>(1.58-1.50)	50.0-1.32<br>(1.37-1.32)	50.0-1.24<br>(1.28-1.24)	50.0-1.36<br>(1.37-1.41)	50.0-1.28<br>(1.32-1.28)	50.0-1.30<br>(1.35-1.30)	50.0-1.32<br>(1.37-1.32)	50.0-1.34<br>(1.39-1.34)
Space group	*I*222	*I*222	*I*222	*I*222	*I*222	*I*222	*I*222	*I*222
Cell dimensions (Å)								
a	79.77	79.51	79.51	79.51	79.50	79.51	79.49	79.50
b	95.13	94.99	95.14	95.16	95.14	95.17	95.14	95.16
c	104.31	104.36	104.35	104.37	104.35	104.40	104.39	104.42
Unique reflections	62422<br>(9106)	92669<br>(8999)	111641<br>(10885)	84909<br>(8246)	101601<br>(9919)	97100<br>(9480)	92728<br>(9004)	88740<br>(8617)
Overall redundancy	3.6<br>(3.5)	6.6<br>(6.6)	6.5<br>(6.1)	6.6<br>(6.6)	6.5<br>(6.2)	6.5<br>(6.6)	6.5<br>(6.6)	6.5<br>(6.6)
Completeness, (%)	98.5<br>(99.4)	100.0<br>(99.9)	99.9<br>(99.8)	100.0<br>(99.9)	99.9<br>(99.9)	99.9<br>(99.9)	100.0<br>(99.9)	100.0<br>(99.9)
R_{merge}, (%)	10.4<br>(55.6)	11.4<br>(71.6)	10.2<br>(72.1)	11.7<br>(72.5)	10.3<br>(69.7)	10.5<br>(75.0)	10.5<br>(73.0)	10.5<br>(72.8)
R_{pim}, (%)	5.9<br>(33.7)	4.8<br>(30.0)	4.3<br>(31.6)	5.0<br>(30.4)	4.4<br>(30.2)	4.5<br>(31.5)	4.5<br>(30.5)	4.5<br>(30.5)
$\langle I/\sigma(I) \rangle$	7.3<br>(2.3)	8.8<br>(2.0)	9.3<br>(2.0)	8.8<br>(2.0)	9.5<br>(2.1)	9.3<br>(2.0)	9.3<br>(2.1)	9.4<br>(2.1)
PDB code	4cw0	4cw2	-	-	4cw6	-	-	4cw3
MUA occupancy	100	-	-	-	-	-	-	-
MUA•+ MUA* occupancy (%)	-	0	22	45	60	71	77	85
5-PMUA occupancy (%)	-	100	78	55	40	29	23	15
O_2 occupancy (%)	-	0	19	38	49	54	57	60
R_{factor}(%)/R_{free}(%)	11.65/15.87	11.04/13.70	10.4/12.64	11.26/13.76	11.21/13.21	10.98/13.69	11.10/13.12	10.92/13.40

**Burn data collections (Figure S4) were performed with flux (photons/s) values of 8.13E10 (B1,B2), 2.44E11 (B3, B4),1.20E12 (B5)*

continued…

Data set	UOX:UA Anaerobic	UOX:5-PIU DC1	UOX:5-PIU DC2	UOX:5-PIU DC3	UOX:5-PIU DC4	UOX:5-PIU DC5	UOX:5-PIU DC6	UOX:5-PIU DC7
Source	in-house	I02/DLS	I02/DLS	I02/DLS	I02/DLS	I02/DLS	I02/DLS	I02/DLS
Crystal size (μm^3)	-	400x400x400	400x400x400	400x400x400	400x400x400	400x400x400	400x400x400	400x400x400
Beam FWHM, vxh (μm^2)	-	21x82	21x82	21x82	21x82	21x82	21x82	21x82
Flux (photons/s)**	-	1.40E10	1.40E10	1.40E10	1.40E10	1.40E10	1.40E10	1.40E10
Total exposure time (s)		15.0	15.0	15.0	15.0	15.0	15.0	15.0
Diffraction weighted dose (kGy)	-	2.2	10	34	57	106	155	204
Wavelength (Å)	1.54	0.97625	0.97625	0.97625	0.97625	0.97625	0.97625	0.97625
Resolution range (Å)<br>Highest res. Bin (Å)	16.53-1.40<br>(1.48-1.40)	50.0-1.30<br>(1.33-1.30)	52.06-1.29<br>(1.32-1.29)	52.06-1.30<br>(1.33-1.30)	52.06-1.30<br>(1.33-1.30)	52.06-1.30<br>(1.33-1.30)	52.06-1.30<br>(1.33-1.30)	52.06-1.30<br>(1.33-1.30)
Space group	*I*222	*I*222	*I*222	*I*222	*I*222	*I*222	*I*222	*I*222
Cell dimensions (Å)								
a	78.92	79.93	79.93	79.93	79.93	79.93	79.93	79.93
b	94.87	95.080	95.08	95.08	95.08	95.08	95.09	95.09
c	104.15	104.06	104.06	104.06	104.06	104.06	104.07	104.07
Unique reflections	76756<br>(11060)	96758<br>(7033)	98985<br>(7195)	96767<br>(7032)	96770<br>(7033)	96765<br>(7038)	96804<br>(7053)	96802<br>(7049)
Overall redundancy	3.6<br>(2.4)	6.6<br>(6.3)	6.6<br>(6.2)	6.6<br>(6.3)	6.6<br>(6.3)	6.6<br>(6.3)	6.6<br>(6.3)	6.6<br>(6.3)
Completeness, (%)	99.8<br>(99.5)	99.6<br>(98.6)	99.6<br>(98.5)	99.6<br>(98.6)	99.6<br>(98.6)	99.6<br>(98.7)	99.6<br>(98.9)	99.6<br>(98.8)
R_{meas}, (%)	5.0<br>(32.6)	10.1<br>(62.4)	10.3<br>(62.5)	10.2<br>(61.7)	10.1<br>(61.9)	10.1<br>(62.9)	10.1<br>(63.3)	10.2<br>(64.3)
R_{pim}, (%)	2.9<br>(26.3)	4.7<br>(29.6)	4.7<br>(30.3)	4.7<br>(29.5)	4.7<br>(29.6)	4.7<br>(30.0)	4.7<br>(30.2)	4.7<br>(30.6)
$\langle I/\sigma(I) \rangle$	14.9<br>(2.4)	9.8<br>(2.4)	9.6<br>(2.4)	9.7<br>(2.5)	9.7<br>(2.5)	9.7<br>(2.4)	9.7<br>(2.4)	9.7<br>(2.4)
PDB code	4d12	4d13	-	-	-	4d17	-	-
UA occupancy	100	-	-	-	-	-	-	-
UA•+ UA* occupancy (%)	-	0	19	30	37	46	51	60
5-PIU occupancy (%)	-	100	81	70	63	54	49	40
O_2 occupancy (%)	-	0	16	27	30	37	44	48
R_{factor}(%)/R_{free}(%)	11.78/14.19	10.76/13.16	10.80/13.13	10.86/13.10	10.79/13.05	10.84/13.08	10.80/12.69	10.84/13.18

***Burn data collections (Figure S5) were performed with flux (photons/s) values of 1.4E10 (B1), 7.0E10 (B2,B3), 1.6E11 (B4,B5, B6), 9.0E11 (B7, B8), 1.82E12 (B9, B10)*

continued…

Data set	UOX:5-PIU DC8	UOX:5-PIU DC9	UOX:5-PIU DC10	UOX:5-PIU DC11
Source	I02/DLS	I02/DLS	I02/DLS	I02/DLS
Crystal size (μm^3)	400x400x400	400x400x400	400x400x400	400x400x400
Beam FWHM, vxh (μm^2)	21x82	21x82	21x82	21x82
Flux (photons/s)**	1.40E10	1.40E10	1.40E10	1.40E10
Total exposure time (s)	15.0	15.0	15.0	15.0
Diffraction weighted dose (kGy)	461	718	1234	1750
Wavelength (Å)	0.97625	0.97625	0.97625	0.97625
Resolution range (Å)<br>Highest res. Bin (Å)	52.06-1.31<br>(1.34-1.31)	52.06-1.32<br>(1.35-1.32)	52.06-1.37<br>(1.37-1.34)	52.06-1.35<br>(1.39-1.35)
Space group	*I*222	*I*222	*I*222	*I*222
Cell dimensions (Å)<br>a<br>b<br>c	 79.93<br>95.10<br>104.08	 79.94<br>95.11<br>104.09	 79.94<br>95.12<br>104.10	 79.94<br>95.13<br>104.11
Unique reflections	94653<br>(6860)	92608<br>(6757)	86620<br>(6488)	86674<br>(6272)
Overall redundancy	6.6<br>(6.6)	6.6<br>(6.6)	6.6<br>(6.7)	6.6<br>(6.7)
Completeness, (%)	99.6<br>(98.9)	99.6<br>(98.9)	98.7<br>(97.9)	99.7<br>(99.0)
R_{meas}, (%)	10.1<br>(64.5)	10.0<br>(63.1)	9.8<br>(62.6)	9.8<br>(65.3)
R_{pim}, (%)	4.7<br>(30.2)	4.6<br>(29.3)	4.5<br>(28.4)	4.5<br>(29.7)
$\langle I/\sigma(I) \rangle$	9.8<br>(2.4)	9.9<br>(2.5)	10.3<br>(2.6)	10.3<br>(2.5)
PDB code	-	-	-	4d19
UA occupancy	-	-	-	-
UA•+ UA* occupancy (%)	68	71	74	77
5-PIU occupancy (%)	32	29	26	23
O_2 occupancy (%)	52	53	51	52
R_{factor} (%)/R_{free}(%)	10.80/12.76	10.77/12.92	10.64/12.81	10.63/12.97

Table S2. Experimental and theoretical peroxide geometries. Selected 5-PMUA and 5-PIU distances and angles are compared to those of 5-PMUA theoretical models calculated at the MP2/6-31+G* level of theory. Four different valence states within the protein environment were considered. 5-PMUA peroxide with a protonated hydroxyl group (charge = -1) is not reported as it is 62 kcal/mol less stable than the isoelectronic 5-PMUA hydoperoxide (charge = -1). Overall, the best geometry agreement between experiment and theory is for 5-PMUA hydoperoxide (charge = -1).

	Experimental 5-PMUA (5-PIU)	Theoretical 5-PMUA hydoperoxide (charge = -1) 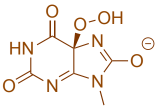	Theoretical 5-PMUA dianion (charge = -2) 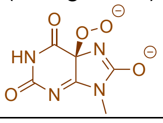	Theoretical 5-PMUA hydroperoxide (charge = 0) 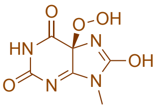
Distances (Å)				
C5-Op1	1.52 (1.51)	1.49	1.45	1.45
Op1-Op2	1.47 (1.47)	1.46	1.48	1.47
N1-C2	1.40 (1.39)	1.40	1.40	1.40
C2-N3	1.41 (1.37)	1.39	1.37	1.39
N3-C4	1.28 (1.32)	1.31	1.32	1.31
C4-C5	1.52 (1.49)	1.51	1.51	1.51
C5-C6	1.52 (1.51)	1.52	1.51	1.52
C6-N1	1.36 (1.37)	1.38	1.39	1.40
C5-N7	1.43 (1.42)	1.40	1.42	1.35
N7-C8	1.38 (1.35)	1.35	1.32	1.46
C8-N9	1.40 (1.39)	1.46	1.49	1.33
N9-C4	1.35 (1.34)	1.33	1.32	1.37
C2-O2	1.22 (1.22)	1.24	1.25	1.22
C6-O6	1.22 (1.23)	1.23	1.23	1.23
C8-O8	1.24 (1.24)	1.24	1.25	1.33
Tetrahedral angles (°)				
C4-C5-Op1	104.04 (103.88)	106.77	106.88	109.31
C6-C5-Op1	94.77 (94.05)	96.86	100.31	99.05
N7-C5-Op1	115.04 (113.99)	114.27	114.37	112.71
C4-C5-C6	110.91 (113.63)	111.18	110.36	110.98
C4-C5-N7	108.58 (108.04)	106.60	105.88	105.45
C6-C5-N7	121.72 (121.41)	120.37	118.58	119.16
Ring angles (°)				
N1-C2-N3	120.08 (122.04)	120.56	120.99	119.83
C2-N3-C4	115.51 (115.39)	116.10	115.36	116.05
N3-C4-C5	127.48 (125.49)	125.18	126.12	126.24
C5-C6-N1	112.98 (113.63)	111.65	112.28	111.55
C6-N1-C2	126.59 (125.81)	125.85	125.96	125.96
C5-N7-C8	103.73 (103.37)	106.70	106.60	103.76
N7-C8-N9	111.16 (113.31)	110.23	109.68	117.43
C8-N9-C4	111.82 (108.79)	107.82	107.68	104.86
N9-C4-C5	101.98 (104.63)	105.99	105.98	105.61

References

[1] O. B. Zeldin, M. Gerstel, E. F. Garman, *Journal of synchrotron radiation* **2013**, *20*, 49-57.

[2] R. L. Owen, E. Rudino-Pinera, E. F. Garman, *Proc Natl Acad Sci U S A* **2006**, *103*, 4912-4917.

[3] M. Weik, R. B. Ravelli, G. Kryger, S. McSweeney, M. L. Raves, M. Harel, P. Gros, I. Silman, J. Kroon, J. L. Sussman, *Proc Natl Acad Sci U S A* **2000**, *97*, 623-628.

[4] P. Emsley, K. Cowtan, *Acta crystallographica. Section D, Biological crystallography* **2004**, *60*, 2126-2132.

[5] G. N. Murshudov, P. Skubak, A. A. Lebedev, N. S. Pannu, R. A. Steiner, R. A. Nicholls, M. D. Winn, F. Long, A. A. Vagin, *Acta crystallographica. Section D, Biological crystallography* **2011**, *67*, 355-367.

[6] P. V. Afonine, R. W. Grosse-Kunstleve, N. Echols, J. J. Headd, N. W. Moriarty, M. Mustyakimov, T. C. Terwilliger, A. Urzhumtsev, P. H. Zwart, P. D. Adams, *Acta crystallographica. Section D, Biological crystallography* **2012**, *68*, 352-367.

[7] G. M. Sheldrick, T. R. Schneider, *Methods in enzymology* **1997**, *277*, 319-343.

[8] P. D. Adams, P. V. Afonine, G. Bunkoczi, V. B. Chen, N. Echols, J. J. Headd, L. W. Hung, S. Jain, G. J. Kapral, R. W. Grosse Kunstleve, A. J. McCoy, N. W. Moriarty, R. D. Oeffner, R. J. Read, D. C. Richardson, J. S. Richardson, T. C. Terwilliger, P. H. Zwart, *Methods* **2011**, *55*, 94-106.

[9] P. Kuzmic, *Methods in enzymology* **2009**, *467*, 247-280.

[10] P. Carpentier, A. Royant, J. Ohana, D. Bourgeois, *Journal of applied crystallography* **2007**, *40*, 1113-1122.

[11] a) B. R. Brooks, C. L. Brooks, 3rd, A. D. Mackerell, Jr., L. Nilsson, R. J. Petrella, B. Roux, Y. Won, G. Archontis, C. Bartels, S. Boresch, A. Caflisch, L. Caves, Q. Cui, A. R. Dinner, M. Feig, S. Fischer, J. Gao, M. Hodoscek, W. Im, K. Kuczera, T. Lazaridis, J. Ma, V. Ovchinnikov, E. Paci, R. W. Pastor, C. B. Post, J. Z. Pu, M. Schaefer, B. Tidor, R. M. Venable, H. L. Woodcock, X. Wu, W. Yang, D. M. York, M. Karplus, *Journal of computational chemistry* **2009**, *30*, 1545-1614; b) S. Jo, T. Kim, V. G. Iyer, W. Im, *Journal of computational chemistry* **2008**, *29*, 1859-1865.

[12] J. C. Phillips, R. Braun, W. Wang, J. Gumbart, E. Tajkhorshid, E. Villa, C. Chipot, R. D. Skeel, L. Kale, K. Schulten, *Journal of computational chemistry* **2005**, *26*, 1781-1802.

[13] Y. Shao, L. F. Molnar, Y. Jung, J. Kussmann, C. Ochsenfeld, S. T. Brown, A. T. Gilbert, L. V. Slipchenko, S. V. Levchenko, D. P. O'Neill, R. A. DiStasio, Jr., R. C. Lochan, T. Wang, G. J. Beran, N. A. Besley, J. M. Herbert, C. Y. Lin, T. Van Voorhis, S. H. Chien, A. Sodt, R. P. Steele, V. A. Rassolov, P. E. Maslen, P. P. Korambath, R. D. Adamson, B. Austin, J. Baker, E. F. Byrd, H. Dachsel, R. J. Doerksen, A. Dreuw, B. D. Dunietz, A. D. Dutoi, T. R. Furlani, S. R. Gwaltney, A. Heyden, S. Hirata, C. P. Hsu, G. Kedziora, R. Z. Khalliulin, P. Klunzinger, A. M. Lee, M. S. Lee, W. Liang, I. Lotan, N. Nair, B. Peters, E. I. Proynov, P.

A. Pieniazek, Y. M. Rhee, J. Ritchie, E. Rosta, C. D. Sherrill, A. C. Simmonett, J. E. Subotnik, H. L. Woodcock, 3rd, W. Zhang, A. T. Bell, A. K. Chakraborty, D. M. Chipman, F. J. Keil, A. Warshel, W. J. Hehre, H. F. Schaefer, 3rd, J. Kong, A. I. Krylov, P. M. Gill, M. Head-Gordon, *Physical chemistry chemical physics : PCCP* **2006**, *8*, 3172-3191.
[14] C. M. Breneman, K. B. Wiberg, *Journal of computational chemistry* **1990**, *11*, 361-373.
[15] M. J. Frisch, G. W. Trucks, H. B. Schlegel, G. E. Scuseria, M. A. Robb, J. R. Cheeseman, G. Scalmani, V. Barone, B. Mennucci, G. A. Petersson, H. Nakatsuji, M. Caricato, X. Li, H. P. Hratchian, A. F. Izmaylov, J. Bloino, G. Zheng, J. L. Sonnenberg, M. Hada, M. Ehara, K. Toyota, R. Fukuda, J. Hasegawa, M. Ishida, T. Nakajima, Y. Honda, O. Kitao, H. Nakai, T. Vreven, J. A. Montgomery, Jr.; , J. E. Peralta, F. Ogliaro, M. Bearpark, J. J. Heyd, E. Brothers, K. N. Kudin, V. N. Staroverov, R. Kobayashi, J. Normand, K. Raghavachari, A. Rendell, J. C. Burant, S. S. Iyengar, J. Tomasi, M. Cossi, N. Rega, N. J. Millam, M. Klene, J. E. Knox, J. B. Cross, V. Bakken, C. Adamo, J. Jaramillo, R. Gomperts, R. E. Stratmann, O. Yazyev, A. J. Austin, R. Cammi, C. Pomelli, J. W. Ochterski, R. L. Martin, K. Morokuma, V. G. Zakrzewski, G. A. Voth, P. Salvador, J. J. Dannenberg, S. Dapprich, A. D. Daniels, Ö. Farkas, J. B. Foresman, J. V. Ortiz, J. Cioslowski, D. J. Fox, Gaussian Inc. , Wallingford CT, **2009**.
[16] O. B. Zeldin, S. Brockhauser, J. Bremridge, J. M. Holton, E. F. Garman, *Proc Natl Acad Sci U S A* **2013**.
[17] M. G. Simic, S. V. Jovanovic, *J Am Chem Soc* **1989**, *111*, 5778-5782.
